# Impacts of dietary calcium, phytate, and phytase on inositol hexakisphosphate degradation and inositol phosphate release in different segments of digestive tract of broilers

**DOI:** 10.3382/ps/pex170

**Published:** 2017-08-02

**Authors:** W. Li, R. Angel, S.-W. Kim, K. Brady, S. Yu, P. W. Plumstead

**Affiliations:** * Department of Animal and Avian Sciences, University of Maryland, College Park, MD 20714, USA; † Enzyme R&D, DuPont Industrial Biosciences, Aarhus, DK-8220, Denmark; ‡ Danisco Animal Nutrition, DuPont Industrial Biosciences, Marlborough, SN8 1AA, UK

**Keywords:** inositol phosphate, digestive tract, phytase, calcium, phytate

## Abstract

A total of 720 straight-run Heritage 56 M × fast feathering Cobb 500F broiler chickens was fed from 11 to 13 d of age to determine the impacts of dietary calcium (Ca), phytate phosphorus (PP), and phytase concentrations on inositol phosphate (IP3–6) profile in different digestive tract (GI) segments. The experiment was a 2 × 2 × 3 randomized block design with 2 Ca (0.7 and 1.0%) and 2 PP (0.23 and 0.34%) concentrations and 3 doses of *Buttiauxella* sp. phytase (0, 500, and 1,000 FTU/kg). The experiment was replicated in time (block) with 3 replicates per treatment (Trt) of 10 birds per block. Concentrations of IP3–6 in the crop, proventriculus (Prov) plus (+) gizzard (Giz), and distal ileum, as well as the ileal IP6 and P disappearance were determined at 13 d of age. The detrimental impact of Ca on IP6 and P disappearance was observed only in the ileum, where 11% reduction in both IP6 and P disappearance was seen when Ca increased from 0.7 to 1.0% (*P* < 0.05). Higher IP5 and IP6 concentrations were seen in both the crop and Prov+Giz at 0.34% PP as compared to birds fed to 0.23% PP diets, regardless of Ca or phytase (*P* < 0.05), whereas IP3 and IP4 concentrations were not affected by PP (*P* > 0.05). Inclusion of phytase, at both 500 and 1,000 FTU/kg, resulted in lower IP6 and the accumulation of lower IP ester (IP3–5) concentrations in all GI segments (*P* < 0.05). Improved IP6 and P disappearance was seen as a result of phytase inclusion, despite the degree of improvement affected by PP (*P* < 0.05). On average, 5.5 and 6.7 times improvement in IP6 was observed with 500 and 1,000 FTU phytase/kg inclusion, respectively, resulting in 41 and 64% greater P digestibility, respectively. In conclusion, phytase can effectively degrade IP6 to lower esters and increase P utilization. However, the efficacy of phytase can be affected by diet Ca and PP concentrations.

## INTRODUCTION

Dietary inclusion of microbial phytase has become an increasingly accepted practice to enhance phytate phosphorus (**PP**) utilization, which is poorly available to poultry species under practical diet nutrient compositions (Amerah et al., 2014). The effectiveness of phytase is usually determined under P or both calcium (**Ca**)- and P-deficient conditions. Phytase efficacy values for a potential increase in available or digestible P and/or Ca are usually given without considering the impact of other dietary factors. Negative impacts of Ca on P and inositol hexakisphosphate (**IP6**) disappearance have been reported in several studies (Tamim and Angel, 2003; Tamim et al., 2004; Ravindran et al., 2000). In addition, Ravindran et al. (2000) reported that the effectiveness of phytase on IP6 and P disappearance was affected by diet PP concentration, where reduced IP6 disappearance was seen with the increase in diet PP from rice bran. However, the negative impact of PP can be alleviated by a high dose of phytase (1,000 FTU/kg), suggesting that the extent of PP effect on IP6 degradation can be manipulated by phytase. Phytase use in poultry is one of the most studied and published areas in the last 20 yr, and yet the implications of the interactions among Ca, PP, and phytase concentrations on phytase efficacy are not clearly understood.

Previously, the impact of dietary factors, including Ca, nonphytate P, and PP, on IP6 degradation was determined in different gastro-intestinal tract (**GIT**) segments of broilers, in the presence and absence of phytase (Li et al., 2016). To follow up previous findings (Li et al., 2016) and allow for a deeper understanding of the dietary impacts on IP esters production, all the digesta samples, except treatments with 0.45% non-phytate P (**nPP**) were further analyzed for lower IP esters, including IP2–5, to determine the impact of diet Ca and PP concentrations at different phytase doses on 1) IP6 and lower IP ester concentrations in the crop, proventriculus plus gizzard (**Prov.+Giz.**), and ileum; and on 2) apparent IP6 and P disappearance in the distal ileum.

## MATERIALS AND METHODS

### Animals and Housing

All animal care procedures were approved by the University of Maryland Animal Care and Use Committee.

The experiment was conducted twice (block) in time with 3 replicates of each treatment (**Trt**) represented in each block. For each block, day-old straight-run Heritage 56 M × fast feathering Cobb 500F broiler chickens were obtained from a local commercial hatchery on d of hatch and placed in floor pen rooms with artificial light and temperature control. A commercial type starter diet, formulated to contain 22.6% CP, 1.2% digestible Lys, 3,010 kcal/kg ME, 1.0% Ca, and 0.48% nPP that met or exceeded all NRC (1994) recommendations as well as average nutrient usage concentrations in the United States for 2012 (AgriStats 2013, end of year summary for 2012), was fed from hatch to d 10. On the morning of d 11, birds were individually weighed, grouped (10 birds/group) such that all groups had similar weight and within-group chick weight variation was minimized, and placed into battery pens (Modified Petersime grower batteries, Petersime Incubator Co, Gettysburg OH) preassigned to treatment (**Trt**). The wire-floored battery pens (width × depth × height; 99 cm × 68 cm × 37 cm) were equipped with nipple drinkers (2 per pen) and 2 external feed troughs (length × width × depth; 63.5 cm × 8.9 cm × 5.67 cm). On the morning of d 13 (48 h), all birds in a pen were euthanized by cervical dislocation, and contents from the crop, Prov+Giz, and distal ileum were sampled, as described in the sampling section of the paper, from every bird and pooled by pen and intestinal section.

The photoperiod was 24 light **(L)**:0 dark **(D)** from hatch to 3 d, 16L:8D from 4 to 7 d, and 20L:4D from 8 to 11 d of age. Room temperature was kept at an average of 32°C from hatch to 3 d with brooder lamps providing additional heat. Temperature was lowered by 1°C every 2 to 3 d such that bird comfort was maintained, and temperature was 29°C at 11 d of age. Birds were checked twice daily, and weights of the dead birds, the remaining birds in the pen, and of the feed were recorded. Feed and water were offered for ad libitum consumption throughout the trial.

### Experimental Design and Diets

Two corn and SBM based mash basals^1^ with either low^2^ [geometric mean diameter (**d_gw_**) = 0.647 mm; standard deviation (**S_gw_**) = 0.719 mm] or high^3^ (**d_gw_**= 0.697; **S_gw_**= 0.731) in PP were formulated based on analyzed ingredient compositions (dry matter, ash, fat, amino acid, Ca, and total P and PP), mixed and analyzed for dry matter, macro minerals, protein, ether extract, and amino acids (Table 1). Meat meal (5.07%) and rice bran (6.00%) were included in the low and high PP basals, respectively, to achieve the desired differences in PP concentration while maintaining similar concentrations of other nutrients. Based on analyzed Ca and P concentrations in the basal diets, pre-analyzed limestone^4^ (IMI Cal Pro, IN; **d_gw_** = 0.402 mm; **S_gw_** = 0.255 mm) and monocalcium phosphate^5^ (Kirby Agri, PA; d_gw_ = 0.759 mm; S_gw_ = 0.258 mm) were added to achieve desired Ca and nPP concentrations in Trt diets. The basal containing either low or high PP was included at 96.7% in the final diets, titanium dioxide (**TiO_2_**) was added at 0.3% as the inert marker, and Celite^®^ (Imerys Filtration Minerals, San Jose, CA) was used as a filler to achieve 100%. The experiment was a 2 × 2 × 3 randomized block design with 2 Ca (0.7 and 1.0%), 2 PP (0.23 and 0.34%), and 3 phytase (0, 500, and 1,000 FTU/kg; Axtra^®^ PHY, Danisco Animal Nutrition, DuPont Industrial Biosciences, Marlborough, UK) concentrations, resulting in a total of 24 Trts (Table 2). Each Trt was replicated 3 times in each of 2 blocks, and the replicate was a pen with 10 birds, resulting in 6 replicates per Trt. Each diet without phytase was divided into 3 lots, and a 6-phytase (derived from *Buttiauxella* sp. and expressed in *Trichodema*, Danisco Animal Nutrition, DuPont Industrial Biosciences, Marlborough, UK) was added on top, at 0, 500 or 1,000 FTU/kg, to one of the 3 lots of the Trt and mixed so that the only difference among those lots was the phytase concentration. The starter and Trt diets were fed as mash form throughout the trial.

**Table 1.** Ingredient and chemical composition of the basal diets.

	Basal (%, as-fed basis)	
Ingredient	Low PP	High PP
Corn	62.82	53.45
Soybean meal (48% CP)	28.5	35.10
Meat meal	5.07	–
Rice bran	–	6.00
Soy oil	1.70	2.79
Salt	0.46	0.50
DL-Methionine, 99%	0.34	0.35
Biolys, 55%	0.57	0.44
L-Threonine, 98.5%	0.19	0.19
Choline chloride, 25%	0.19	0.18
Mineral premix^1^	0.08	0.08
Vitamin premix^2^	0.08	0.08
Limestone (36.5%)^3^	–	0.45
Monocalcium phosphate^4^	–	0.39
Total	100.00	100.00
Calculated (analyzed, mean±SD) concentrations (%)		
ME_n_, kcal/kg	3,100	3,100
Crude protein	22.5 (23.9 ± 0.8)	22.5 (25.9 ± 1.1)
Lysine	1.41 (1.43 ± 0.01)	1.41 (1.40 ± 0.04)
Methionine + Cystine	1.07 (1.06 ± 0.03)	1.05 (1.05 ± 0.03)
Calcium (Ca)	0.49 (0.50 ± 0.02)	0.49 (0.50 ± 0.02)
Total phosphorus (P)	0.48 (0.52 ± 0.02)	0.59 (0.62 ± 0.01)
Phytate phosphorus (PP)	0.23 (0.25 ± 0.00)	0.34 (0.34 ± 0.00)
Non-phytate phosphorus (nPP)^5^	0.25 (0.27)	0.25 (0.28)
Inositol phosphate (IP)		
IP6	0.82 (0.89 ± 0.00)	1.22 (1.22 ± 0.00)
IP5	(0.078 ± 0.03)	(0.115 ± 0.01)
IP3 and 4	Not detected	Not detected

^1^Supplied per kg of diet: zinc from zinc sulfate, 85 mg; manganese from manganese sulfate, 107 mg; iron from iron sulfate, 21 mg; magnesium from magnesium oxide, 21 ppm; selenium from selenium sulfate, 0.32 ppm; copper from copper sulfate, 3 mg; iodine from calcium iodate, 4 mg.

^2^Supplied per kg of diet: vitamin A, 13,151 IU; vitamin D, 4642 IU; vitamin E, 46.42 IU; vitamin B_12_, 0.02 mg; riboflavin, 15 mg; niacin, 62 mg; pantothenic acid, 21.7 mg; vitamin K_3_, 2.8 mg; folic acid, 1.87 mg; biotin, 0.13 mg; thiamine, 4 mg; pyridoxine, 5.4 mg.

^3^IMI Cal Pro, Irving Materias, IN. Analyzed Ca, 36.65%.

^4^Analyzed Ca and P: 16.06 and 21.95%, respectively.

^5^Concentration determined based on analyzed *P*-value minus analyzed PP.

**Table 2.** Formulated (calculated) and analyzed (determined) calcium (Ca), total phosphorus (P), phytate P (PP), nonphytate P (nPP), and phytase concentrations (mean±SD) in final diets (as is basis).^1^

Ca, %		P, %		PP^1^, %		nPP, %		Phytase, FTU/kg	
Fml^2^	Ana^2^	Fml	Ana	Fml	Ana	Cal^2^	Det^2^	Fml	Ana
0.7	0.70 ± 0.00	0.51	0.52 ± 0.00	0.23	0.24	0.28	0.28	0	<50
0.7	0.68 ± 0.00	0.62	0.63 ± 0.00	0.34	0.33	0.28	0.30	0	<50
1	0.89 ± 0.04	0.51	0.51 ± 0.01	0.23	0.24	0.28	0.27	0	<50
1	0.93 ± 0.02	0.62	0.63 ± 0.02	0.34	0.33	0.28	0.30	0	<50
0.7	0.70 ± 0.00	0.51	0.52 ± 0.00	0.23	0.24	0.28	0.28	500	511 ± 162
0.7	0.68 ± 0.00	0.62	0.63 ± 0.00	0.34	0.33	0.28	0.30	500	586 ± 62
1	0.89 ± 0.04	0.51	0.51 ± 0.01	0.23	0.24	0.28	0.27	500	545 ± 150
1	0.93 ± 0.02	0.62	0.63 ± 0.02	0.34	0.33	0.28	0.30	500	546 ± 18
0.7	0.70 ± 0.00	0.51	0.52 ± 0.00	0.23	0.24	0.28	0.28	1000	989 ± 144
0.7	0.68 ± 0.00	0.62	0.63 ± 0.00	0.34	0.33	0.28	0.30	1000	1004 ± 52
1	0.89 ± 0.04	0.51	0.51 ± 0.01	0.23	0.24	0.28	0.27	1000	1010 ± 0
1	0.93 ± 0.02	0.62	0.63 ± 0.02	0.34	0.33	0.28	0.30	1000	1008 ± 159

^1^Calculated as analyzed percent PP in basal × 96.7% (inclusion of high or low PP basal in final diets). Analyzed PP concentrations in low and high PP basals are 0.25 ± 0.00 and 0.34 ± 0.00, respectively.

^2^Fml: formulated concentrations; Ana: analyzed concentrations; CAL: calculated concentrations calculated by the difference between Fml P and Fml PP; Det: determined concentrations.

In the current trial, broiler birds were fed Trt diets for only 2 d, from 11 to 13. From several previous trials conducted in our lab (Tamim and Angel, 2003; Tamim et al., 2004; Proszkowiec-Weglarz et al., 2013), feeding for 48 h minimized the ability of broiler chickens to alter P digestibility when Ca- or P-deficient or P- and Ca-imbalanced diets are fed.

### Sample Collection and Lab Analysis

At 13 d of age, all birds within a pen were sacrificed by cervical dislocation such that removal of the GIT segment was done within 30 seconds. Contents from the crop, Prov+Giz, and ileum from each bird were placed into a container, by intestinal section, maintained in an ice bath until all birds in a pen were sampled, and immediately frozen at −20 °C to prevent any further effects of phytase on IP6. The last half of the ileum (distal half of the ileal segment encompassed between the Meckel's diverticulum and 3 cm from the ileocecal junction) was removed, placed on an ice cold marble slab and the contents gently expressed by flushing with ice-cold, double-distilled water. Digesta contents from different segment of the GIT were pooled by pen and segment-freeze dried, ground (0.25 mm screen), and stored in air-tight containers at 4 °C until analyzed.

Samples were analyzed in duplicate, except where specified otherwise. Dry matter of diets and digesta contents were determined by drying overnight in a 100°C force draft oven (Shreve et al., 2006). Diet and ileal Ca and P were determined, in triplicate, after acid digestion and analyzed using inductively coupled plasma atomic emission spectrometry (**ICP-AES**; AOAC, 1999). Titanium (**Ti**) concentrations in diets and ileal digesta were determined by a colorimetric method adapted from Short et al. (1996) where samples were first ashed at 580°C and then digested in 7.4 M H_2_SO_4._ Crude protein and ether extract in basal diets were analyzed according to AOAC methods 990.03 (2003a) and 920.39 (2003b), respectively. Concentrations of amino acids were predicted by AMINONIR^®^ (Evonik Industries, Kennesaw, USA) in corn, SBM, meat meal, and rice bran and analyzed by AMINOLab^®^ (Evonik Industries, Kennesaw, GA) in the basal diets. Different concentrations of IP esters (IP3–6) in basal diets and digesta were analyzed according to the high-performance ion exchange chromatography method (Yu et al., 2012) adapted from Skoglund et al. (1997) and Skoglund et al. (1998). Phytase activities in all Trt diets were determined, in blind triplicate, according to the ISO 30024 (2009) procedure where one phytase unit (**FTU**) is the amount of enzyme that releases 1μmol of inorganic orthophosphate from a sodium phytate substrate per min at pH 5.5 and 37°C.

### Calculations

Apparent ileal IP6 and P disappearance was calculated based on the following formula using TiO_2_ as the inert marker:

\begin{eqnarray*}
{\rm{Disappearance}} = \frac{{{{({\rm{Con}}/{\rm{TiO}_2})}_{\rm{d}}} - {{({\rm{Con}}/{\rm{TiO}_2})}_{\rm{i}}}}}{{{{({\rm{Con}}/{\rm{TiO}_2})}_{\rm{d}}}}} \times 100{\rm{\% }}
\end{eqnarray*}

Where (Con/TiO_2_)_d_ is the ratio of IP6 or P to TiO_2_ in the diet, and (Con/TiO_2_)_i_ is the ratio of IP6 or P to TiO_2_ in the ileal digesta.

Phytase efficacy was calculated as the digestible P increase between birds fed phytase and non-phytase diets with the Ca and PP concentrations:

\begin{eqnarray*}
{\rm DigP\ increase} =
\left( {{\rm{P\ dig}_{{\rm{phy}}}} - {\rm P\ dig}_{{\rm{non}} - {\rm{phy}}}} \right)
\times {{\rm{P}}_{{\rm{diet}}}}
\end{eqnarray*}

Where P_digphy_ is P digestibility in birds fed the phytase diet; P_dignon-phy_, is P digestibility in birds fed the non-phytase diet; and P_diet_ is the analyzed total P concentration in the diet.

### Statistical Analysis

Data were analyzed by the GLM procedure of JMP 12 (SAS Institute, 2015). Trt was considered as a fixed effect and pen within a block as a random effect. Effects of diet Ca, PP, and phytase concentrations and their interactions were determined. Tukey's (Tukey, 1949) adjustment was applied in all pair-wise comparisons to protect *P*-values. Pen within a block was the experimental unit, and all calculations were generated based on pen averages. Significance was declared at *P* < 0.05.

## RESULTS

Analyzed concentrations of Ca, P, phytate, and phytase were all close to formulated values. Phytase activities were below the detection limit in non-phytase Trts. Analyzed phytase activities in phytase Trts were within 10% of formulated values, except in the 0.7% Ca, 0.34% PP containing 500 FTU phytase/kg, where the analyzed phytase concentration was 17% higher than the formulated value (Table 2).

**Figure 1.**
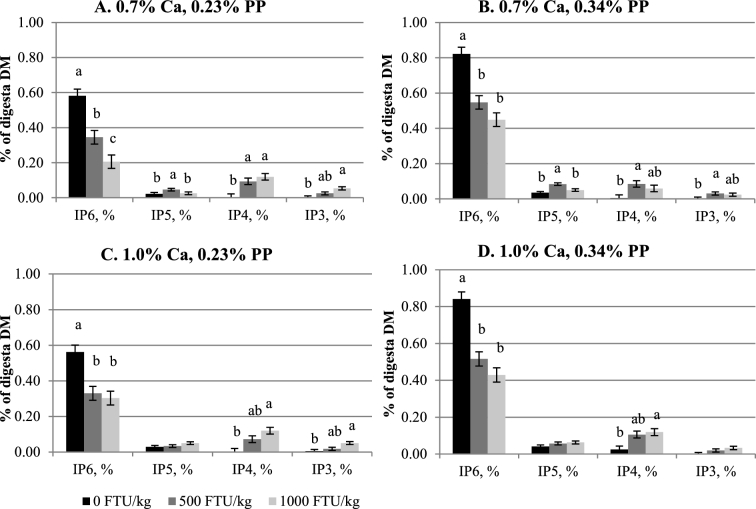
Effect of phytase on inositol phosphate ester (IP3–6) concentrations in crop (mean±SEM). On DM basis, analyzed IP6 and IP5 concentrations were 0.998 and 0.088%, respectively, in low PP basal, and 1.368 and 0.129%, respectively, in high PP basal. No IP3 or 4 concentration was detected in either low or high PP basals. ^a-c^ Comparisons were made within the same IP ester group, using Tukey's test. Means with different superscript letters differ.

**Figure 2.**
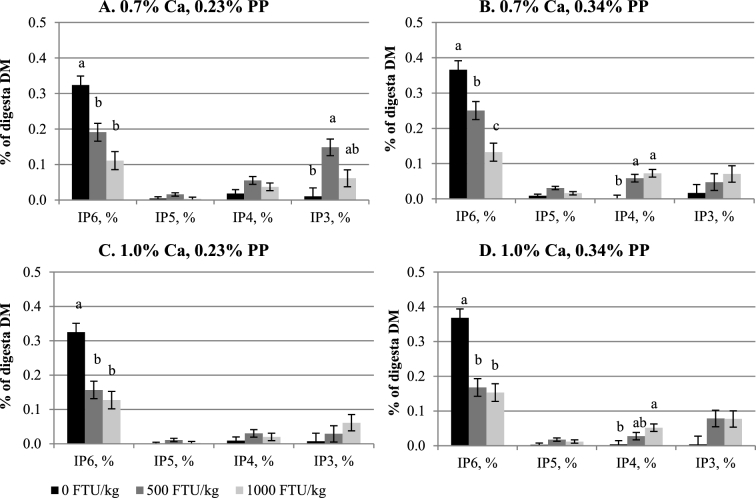
Effect of phytase on inositol phosphate ester (IP3–6) concentrations in proventriculus and gizzard (mean±SEM). On DM basis, analyzed IP6 and IP5 concentrations were 0.998 and 0.088%, respectively, in low PP basal, and 1.368 and 0.129%, respectively, in high PP basal. No IP3 or 4 concentration was detected in either low or high PP basals. ^a-c^ Comparisons were made within the same IP ester group. Means with different superscript letters differ.

**Figure 3.**
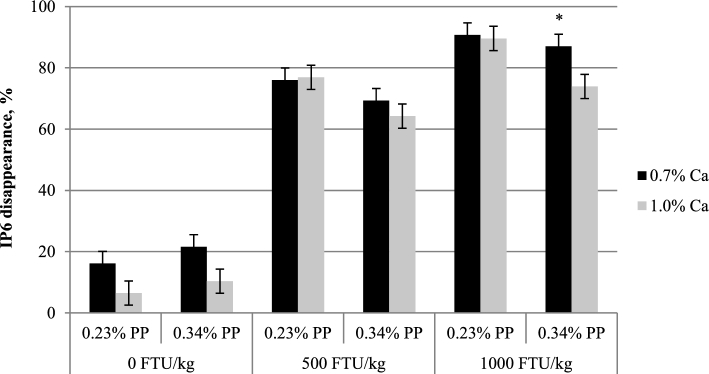
Effects of Ca on ileal IP6 disappearance (mean±SEM). Comparisons were made between birds fed the diets containing the same PP and phytase. * signifies a Ca effect (*P <* 0.05).

**Figure 4.**
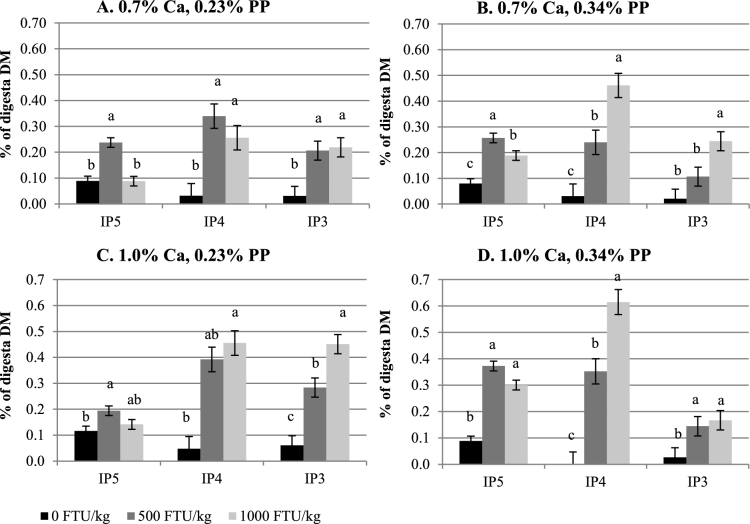
Effect of phytase on ileal inositol phosphate ester (IP3–5) concentrations (mean±SEM). ^a-c^ Comparisons were made within the same IP ester group. Means with different superscript letters differ.

**Figure 5.**
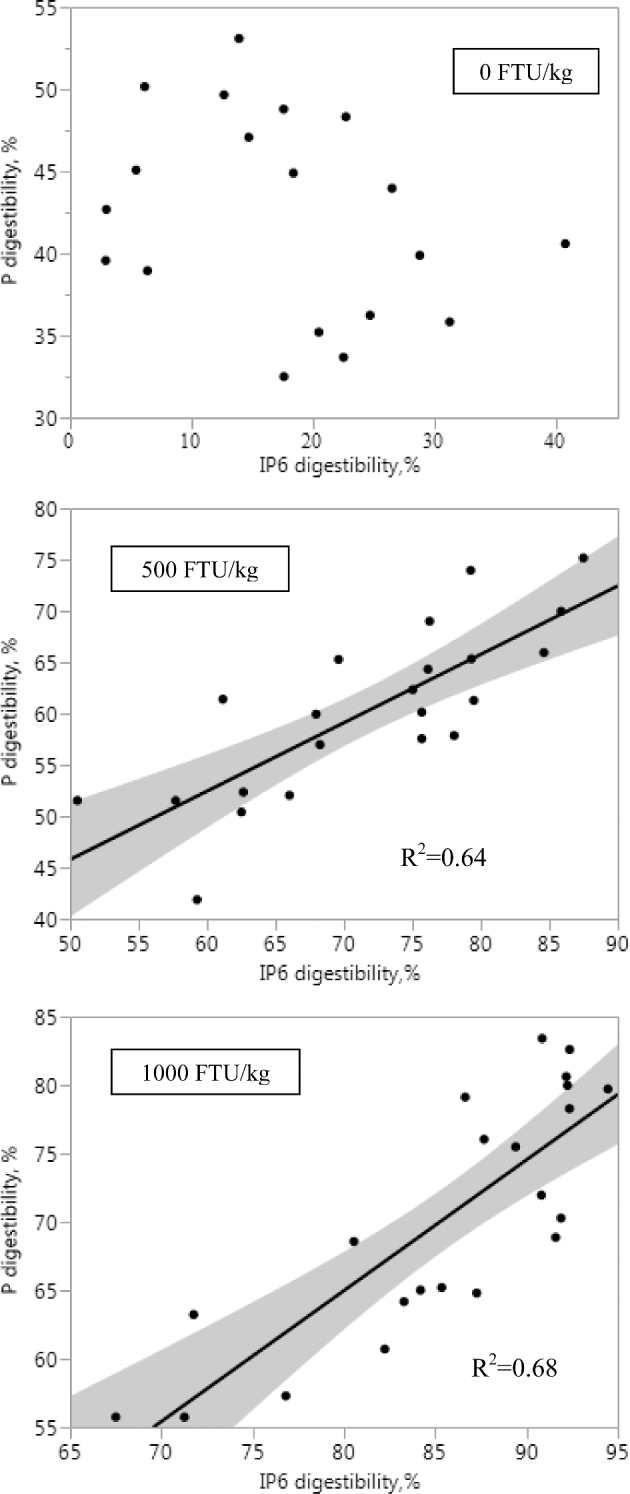
Correlation between ileal inositol hexakisphosphate (IP6) and total P digestibility in the presence and absence of phytase.

Crop IP6 and lower IP ester (IP3–5) concentrations were not affected by Ca (Table 3, Figure 1A-D). Higher IP5 and IP6 concentrations were seen in birds fed 0.34% PP diets as compared to those fed 0.23% PP diets (*P <* 0.05), whereas concentrations of IP3 and IP4 were not influenced by PP. Inclusion of phytase reduced IP6 concentration and consequently increased IP3 and IP4 concentrations (*P <* 0.05), but these effects were not affected by Ca or PP. The only interaction observed (*P <* 0.05) was between Ca and phytase for IP5 concentration. Similarly, diet Ca did not affect IP3, 5, and 6 concentrations in the Prov+Giz (Table 4, Figure 2A-D), except the IP4 concentration was 40% lower in birds fed 1.0% Ca than 0.7% Ca diets (0.024% vs. 0.040%; *P <* 0.05). Prov+Giz IP5 and IP6 concentrations were 16% and 1.5 times higher, respectively, in birds fed 0.34% vs. 0.23% PP diets (*P <* 0.05). No PP effect was seen on IP3 or IP4 concentration. Similar to what was seen in the crop, phytase reduced (*P <* 0.05) IP6 concentration by 45 and 63% when diets with 500 and 1,000 FTU phytase/kg were fed. Unlike in the crop and Prov+Giz, where Ca had no negative effect on concentration of IP6 and its lower esters, increasing diet Ca decreased ileal IP6 and P disappearance and affected concentrations of lower IP esters (Table 5; *P <* 0.05). Impact of PP on IP6 disappearance was affected by phytase. Phytate-P had no effect on IP6 disappearance in the absence of phytase, whereas reduced IP6 disappearance was seen as a result of increasing PP (Figure 3; *P <* 0.05) when phytase was fed. Ileal IP6 disappearance was improved (*P <* 0.05) 5.5 and 6.7 times as a result of the dietary inclusion of 500 and 1,000 FTU phytase/kg, respectively. Similar to results seen in the upper part of the digestive tract, there was an accumulation of the lower IP esters in the ileum of birds fed diets containing phytase, as compared to those fed non-phytase diets (Figure 4A-D; *P <* 0.05). However, the lower IP ester profile differed depending on diet Ca and PP. There was no correlation between ileal IP6 digestibility in the absence of phytase but when phytase was added at 500 or 1000 FTU/kg the correlation was 0.64 and 0.68 respectively (Figure 5).

**Table 3.** Effects of dietary calcium (Ca), phytate P (PP), nonphytate P (nPP), and phytase concentrations on crop inositol phosphate esters (IP3 to 6) concentrations (% of the digesta DM) in birds fed experimental diets from 11 to 13 d of age.^1^

Ca^2^, %	PP^2^, %	Phytase^2^, FTU/kg	IP6, %	IP5, %	IP4, %	IP3, %
0.70	0.23	0	0.581^b^	0.022^c^	0.003^d^	0.001^b^
0.70	0.23	500	0.345^c-e^	0.046^b,c^	0.093^a-c^	0.025^a,b^
0.70	0.23	1000	0.206^e^	0.025^b,c^	0.119^a^	0.054^a^
0.70	0.34	0	0.821^a^	0.034^b,c^	0.004^c,d^	0.001^b^
0.70	0.34	500	0.547^b^	0.083^a^	0.084^a-d^	0.030^a,b^
0.70	0.34	1000	0.448^b-d^	0.050^a-c^	0.059^b,a,c,d^	0.024^a,b^
1.00	0.23	0	0.562^b^	0.029^b,c^	0.002^d^	0.006^b^
1.00	0.23	500	0.330^d,e^	0.033^b,c^	0.072^a-d^	0.018^a,b^
1.00	0.23	1000	0.303^d,e^	0.050^a-c^	0.120^a^	0.051^a^
1.00	0.34	0	0.841^a^	0.041^b,c^	0.025^b-d^	0.000^b^
1.00	0.34	500	0.516^b,c^	0.058^a,b^	0.106^a,b^	0.020^a,b^
1.00	0.34	1000	0.430^b-d^	0.063^a,b^	0.119^a^	0.033^a,b^
**SEM**			0.0386	0.0076	0.0186	0.0088
***P*-values** **^3^**			<0.001	<0.001	<0.001	<0.001
**Main effect means** **^4^**						
	Ca, %	0.7	0.491	–	0.061	0.023
		1.0	0.497	–	0.074	0.021
		**SEM**	0.0160	–	0.0078	0.0038
	PP, %	0.23	0.388^b^	0.034^b^	0.068	0.026
		0.34	0.601^a^	0.055^a^	0.066	0.018
		**SEM**	0.0160	0.0031	0.0077	0.0037
	Phytase, FTU/kg	0	0.701^a^	–	0.008^b^	0.002^c^
		500	0.435^b^	–	0.089^a^	0.023^b^
		1000	0.347^c^	–	0.104^a^	0.040^a^
		**SEM**	0.0193	–	0.0093	0.0044
***P*-values** **^5^**						
	Ca		0.800	0.613	0.225	0.795
	PP		<.001	<.001	0.852	0.148
	Phytase		<.001	<.001	<.001	<.001
	Ca × PP		0.494	0.347	0.063	0.924
	Ca × Phytase		0.531	0.002	0.520	0.605
	PP × Phytase		0.336	0.229	0.200	0.104
	Ca × PP × Phytase		0.370	0.810	0.782	0.757

^1^n = 6, 2 blocks, 3 n/block, 10 birds/n.

^2^Formulated concentrations, analyzed concentrations are shown in Table 2.

^3^Denotes the overall treatment effect without considering the specific main effect of diet.

^4^Main effects are shown only when there are no interactions.

^5^Denotes the main effect *P*-values.

^a-e^Means within a column with different superscript letters differ (*P* < 0.05).

**Table 4.** Effects of dietary calcium (Ca), phytate P (PP), nonphytate P (nPP), and phytase concentrations on proventriculus and gizzard inositol phosphate esters (IP3–6) concentrations (% of the digesta DM) of birds fed experimental diets from 11 to 13 d of age.^1^

Ca^2^, %	PP^2^, %	Phytase^2^, FTU/kg	IP6, %	IP5, %	IP4, %	IP3, %
0.70	0.23	0	0.324^a^	0.005^b^	0.018^b-d^	0.010^b^
0.70	0.23	500	0.191^b,c^	0.016^a,b^	0.055^a-c^	0.148^a^
0.70	0.23	1000	0.111^c^	0.003^b^	0.037^a,b,c,d^	0.061^a,b^
0.70	0.34	0	0.366^a^	0.009^a,b^	0.000^d^	0.017^b^
0.70	0.34	500	0.250^a,b^	0.031^a^	0.059^a,b^	0.048^a,b^
0.70	0.34	1000	0.132^b,c^	0.016^a,b^	0.073^a^	0.071^a,b^
1.00	0.23	0	0.325^a^	0.000^b^	0.009^b-d^	0.007^b^
1.00	0.23	500	0.157^b,c^	0.011^a,b^	0.031^a-d^	0.029^b^
1.00	0.23	1000	0.127^b,c^	0.002^b^	0.020^b-d^	0.061^a,b^
1.00	0.34	0	0.369^a^	0.003^b^	0.004^c,d^	0.004^b^
1.00	0.34	500	0.168^b,c^	0.017^a,b^	0.028^a-d^	0.078^a,b^
1.00	0.34	1000	0.153^b,c^	0.012^a,b^	0.052^a-d^	0.077^a,b^
**SEM**			0.0254	0.0048	0.0108	0.0236
***P*-values** **^3^**			<0.001	0.003	<0.001	0.001
**Main effect means** **^4^**						
Ca, %		0.7	0.229	0.013	0.040^a^	–
		1.0	0.216	0.008	0.024^b^	–
		**SEM**	0.0105	0.0020	0.0044	–
PP, %		0.23	0.206^b^	0.006^b^	–	–
		0.34	0.240^a^	0.015^a^	–	–
		**SEM**	0.0104	0.0020	–	–
Phytase, FTU/kg		0	0.346^a^	0.004^a^	–	–
		500	0.191^b^	0.019^b^	–	–
		1000	0.131^c^	0.008^b^	–	–
		**SEM**	0.0127	0.0024	–	–
***P*-values** **^5^**						
Ca			0.396	0.054	0.012	0.204
PP			0.026	0.005	0.256	0.762
Phytase			<0.001	<0.001	<0.001	<0.001
Ca × PP			0.623	0.435	0.943	0.061
Ca × Phytase			0.095	0.656	0.260	0.296
PP × Phytase			0.863	0.465	0.014	0.460
Ca × PP × Phytase			0.723	0.856	0.796	0.023

^1^n = 6, 2 blocks, 3 n/block, 10 birds/n.

^2^Formulated concentrations, analyzed concentrations are shown in Table 2.

^3^Denotes the overall treatment effect without considering the specific main effect of diet.

^4^Main effects are shown only when there are no interactions.

^5^Denotes the main effect *P*-values.

^a-d^Means within a column with different superscript letters differ (*P* < 0.05).

**Table 5.** Effects of dietary Ca, phytate P (PP), nonphytate P (nPP), and phytase concentrations on ileal inositol phosphate esters (IP3–6) concentrations and disappearance of total P an IP6 of birds fed experimental diets from 11 to 13 d of age.^1^

			Disappearance, %		Concentration, %		
Ca^2^, %	PP^2^, %	Phytase^2^, FTU/kg	IP6	P	IP5	IP4	IP3
0.70	0.23	0	16.12^d^	46.42^e,f^	0.089^e^	0.032^c,d^	0.031^d,e^
0.70	0.23	500	76.02^a-c^	66.23^a-c^	0.237^b,c^	0.340^b^	0.206^b-d^
0.70	0.23	1000	90.75^a^	76.73^a^	0.088^e^	0.256^b,c^	0.219^b,c^
0.70	0.34	0	21.61^d^	45.72^e,f^	0.080^e^	0.031^c,d^	0.021^e^
0.70	0.34	500	69.28^b,c^	60.42^c,d^	0.257^b,c^	0.240^b,c^	0.106^b-e^
0.70	0.34	1000	86.99^a,b^	70.32^a-c^	0.188^c,d^	0.461^a,b^	0.244^b^
1.00	0.23	0	6.46^d^	39.81^e,f^	0.116^d,e^	0.047^c,d^	0.061^c-e^
1.00	0.23	500	76.92^a-c^	62.77^b,c^	0.194^c,d^	0.392^b^	0.284^a,b^
1.00	0.23	1000	89.58^a^	72.00^a,b^	0.141^d,e^	0.455^a,b^	0.451^a^
1.00	0.34	0	10.35^d^	37.10^f^	0.089^e^	0.000^d^	0.026^e^
1.00	0.34	500	64.24^c^	50.10^d,e^	0.373^a^	0.352^b^	0.145^b-e^
1.00	0.34	1000	73.91^a-c^	58.85^c,d^	0.300^a,b^	0.615^a^	0.167^b-e^
**SEM**			3.962	2.390	0.0186	0.0474	0.0370
***P*-values** **^3^**			<0.001	<0.001	<0.001	<0.001	<0.001
**Main effect means** **^4^**							
Ca, %		0.7	60.13^a^	60.97^a^	–	–	–
		1.0	53.58^b^	53.44^b^	–	–	–
		**SEM**	1.697	0.992	–	–	–
PP, %		0.23	–	–	–	–	–
		0.34	–	–	–	–	–
		**SEM**	–	–	–	–	–
Phytase		0	–	–	–	–	–
FTU/kg		500	–	–	–	–	–
		1000	–	–	–	–	–
		**SEM**	–	–	–	–	–
***P*-values** **^5^**							
Ca			0.008	<0.001	<0.001	0.003	0.021
PP			0.044	<0.001	<0.001	0.287	<0.001
Phytase			<0.001	<0.001	<0.001	<0.001	<0.001
Ca × PP			0.179	0.067	0.004	0.847	0.006
Ca × Phytase			0.356	0.939	0.061	0.029	0.516
PP × Phytase			0.021	0.035	<0.001	0.001	0.089
Ca × PP × Phytase			0.675	0.720	0.006	0.663	0.015

^1^n = 6, 2 blocks, 3 n/block, 10 birds/n.

^2^Formulated concentrations, analyzed concentrations are shown in Table 2.

^3^Denotes the overall treatment effect without considering the specific main effects of diet.

^4^Main effects are shown only when there are no interactions.

^5^Denotes the main effect *P*-values.

^a-f^Means within a column with different superscript letters differ (*P* < 0.05).

## DISCUSSION

Several studies have shown that particle size differences from ingredients have a profound effect on nutrient digestibility (Zhang and Coon, 1997; Charbeneau and Roberson, 2004; Amerah and Ravindran, 2009; Amerah et al., 2014; Anwar et al., 2015), where increased particle size from limestone or other feed ingredient is usually, even though not always, associated with increased digestibility. The current study did not aim to examine the impact of diet and/or ingredient particle size on IP esters degradation. However, providing particle size information for diet and key ingredients will allow more appropriate comparisons for similar work in the future.

The positive effect of phytase on ileal PP and total P digestibilities has been well established in broilers (Sebastian et al., 1998; Angel et al., 2002; Selle and Ravindran, 2007). Recently, there is an increasing interest in looking into the phytase effect in the digesta of the upper digestive tract (crop, proventriculus, and gizzard) phytate degradation, in order to understand better the mechanism and interactions of phytate with other nutrients (Yu et al., 2014; Menezes-Blackburn et al., 2015). In the current study, effects of phytase on IP6 degradation and lower IP ester production were assessed in the crop, Prov+Giz, and ileum in birds fed diets containing different Ca and PP concentrations. Due to the feed/digesta particle size variations in the crop and Prov+Giz, IP6 and its lower ester profiles were expressed on concentration, while disappearances of IP6 and P were calculated in the ileum only (Li et al., 2016).

### IPs Concentrations in the Upper GIT

In agreement with the findings from Walk et al. (2014) and Zeller et al. (2015), substantial reduction in IP6 was seen in both the crop and Prov+Giz as a result of 500 and 1,000 FTU phytase/kg inclusion, regardless of diet Ca or PP concentrations. The same results were also reported by Li et al. (2016), where phytase was found to effectively reduce IP6 concentration, even at high nPP (0.45%) concentrations. These findings confirmed that microbial phytase is highly active in the upper part of the GIT (Yu et al., 2014; Menezes-Blacknurn et al., 2015).

Given the low IP5 detected in the basal diet and that dietary IP 3 and 4 concentrations were below the detection limit, the accumulation of lower IP esters could come only from step-wise degradation of IP6. In both the crop and Prov+Giz, there was little IP5 and minimum IP3 and 4 detected, suggesting that in the absence of phytase, there was little IP6 hydrolysis in these 2 segments of a broiler's GIT. Despite the fast removal of IP6 when phytase was added to the diets, production of lower IP esters differed, depending on phytase dose, Ca, and PP concentrations. It is evident that in those birds fed low PP (0.23%) diets (Figures 1 and 2), IP5 concentration was minimum, but there were significant increases in IP3 (Prv+Giz) and IP4 (crop) concentrations, especially at a high phytase dose (1,000 FTU phytase/kg). These increases in IP3 and 4 suggest that in both GIT segments IP5 was quickly and completely degraded by phytase.

When digesta moved from the crop to Prov+Giz, there was a shift of main IP ester product from IP4 to IP3, indicating IP4 could be further degraded by phytase, and the end product of *Buttiauxella* phytase up to the Prov+Giz is likely to be IP3 and even lower esters, such as IP2 or IP1. Even though in birds fed high PP diets (0.34%) there was more IP5 remaining in both the crop and Prov+Giz, a similar pattern of increases in IP3 concentration was seen at both PP. However, recent (Menezes-Blackburn et al., 2015) in vivo (Walk et al., 2014; Bello et al., 2016) studies showed IP ester production may be different among phytases. For example, Walk et al. (2014) reported that, in broilers fed a Ca and P deficient diet (0.81% Ca and 0.30% available P) with 500, 1,000, and 1,500 FTU *E.coli* phytase/kg inclusion, the major IP ester found in the gizzard was IP4. Even though increasing phytase from 500 to 1,500 FTU/kg resulted in a reduction in IP4 concentration, there was no increase in IP3 at any dose of phytase, as compared to birds fed a non-phytase diet. Similarly, Zeller et al. (2015) also reported that the main IP ester in the gizzard from IP6 hydrolysis was IP4 for *E.coli* phytases, whereas there was no apparent lower IP esters accumulation in the gizzard when *A.niger* phytase was fed to broilers. Differences in the IP ester production patterns can be attributed to the chemical properties of the different phytases, such as the type of phytase, pH optima and range, specific activity, and Km (Menezes-Blackburn et al., 2015). At amino acid level, the *E.coli* phytase shares less than 50% homology with the *Buttiauxella* phytase used in this study (Yu et al., 2014) and less than 20% with *A.niger* phytase.

IP6 solubility, in vitro, can be greatly reduced in the presence of cations (eg. Ca^2+^, Cu^2+^, and Zn^2+^), especially at pH > 4 (Jackman and Black, 1951; Nolan et al., 1987; Tamim et al., 2004). In addition, Persson et al. (1998) pointed out that the chelation between IPs (3–6) and cations was not only pH dependent, but also the affinity towards cations differed among IPs. For example, minimum precipitation was found between Cu^2+^ and all IPs (3–6) at pH < 3.5. The binding capacity between IP esters and Cu^2+^ increased with pH, and IP6 was the most potent molecule followed by IP5, IP4, and IP3 up to pH = 6. Differences in binding capacity among IPs diminished when pH > 6.5, where all IPs (IP3–6) showed similar binding capacity towards Cu^2+^. This in vitro observation is supportive of the lack of Ca effect seen in the upper tract in the current study. Based on the combination of the in vitro and in vivo results, we can hypothesize that any undigested IPs entering the small intestine, where pH will be 6 and above, will have a high chance of binding with divalent cations (Tamin el at., 2004) and thus precipitating, reducing the efficacy of any intestinal phytase (Applegate et al., 2003). Chelation with cations of the remaining IP6 entering the small intestine results in lower P availability from IP6 and reduced cation availability (Tamin et al., 2004). This emphasizes the importance of fast and complete dephosphorylation of IP6 in the upper tract.

### IP6 and P Disappearance and IP3-6 Concentrations in the Distal Ileum

**Figure 6.**
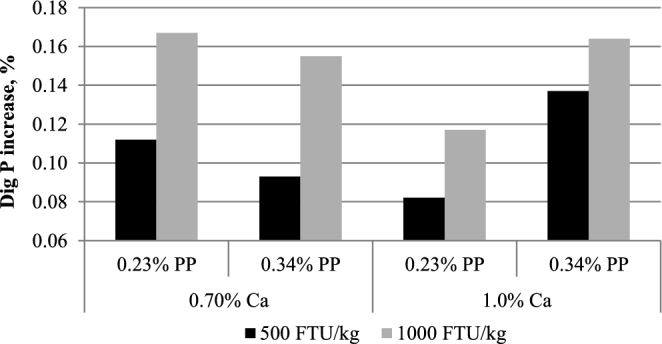
Impacts of Ca and PP on phytase efficacy expressed as digestible P increase. Phytase efficacy was calculated as: Dig P increase (%) = (P_digphy_—P_dignon-phy_) × P_diet_. Dig P increase, net increase in digestible P; P_digphy_, P digestibility in birds fed phytase diets; P_dignon-phy_, P digestibility in birds fed non-phytase diets with same Ca and PP; P_diet_, analyzed total P concentration in the diet.

As a result of effective IP6 removal in upper GIT segments, ileal IP6 and P disappearance was significantly improved in birds fed phytase diets (Figure 3). In addition, in the presence of phytase, ileal P disappearance was highly correlated with IP6 disappearance, where 64 and 68% of the variations in P disappearance were explained by IP6 disappearance at 500 and 1,000 FTU phytase/kg, respectively (Figure 6). However, the interaction between PP and phytase suggests that phytase efficacy also could be influenced by diet PP concentration (Yi et al., 1996; Ravindran et al., 2000; Ravindran et al., 2006). Although dietary IP6 is the sole substrate for phytase, surprisingly very limited research has been done to examine the impact of PP source and/or concentration on phytase efficacy. In broilers fed diets containing a single feed ingredient, including corn, SBM, barley, rice bran, canola meal, wheat or wheat midds, with different concentrations of starch and synthetic amino acids, from hatch to 22 d of age, IP6 hydrolysis was found to be similar among tested ingredients (33%, on average) in the absence of added phytase (Leske and Coon, 1999). Hydrolysis of IP6 was significantly increased due to 600 FTU *A.niger* phytase/kg inclusion, but the degree of improvement depended on ingredient, with the least and greatest improvements seen in rice bran (45%) and barley (121%), respectively. In another study, Ravindran et al. (2000) reported that P degradation could be significantly reduced by increasing phytate concentration, but this negative effect could be alleviated by increasing the concentration of *A.niger* phytase. Thus, phytate source and concentration can interfere with phytase efficacy in vivo. In the current study, ileal IP6 and P disappearance when phytase was included in the diet also was found to be affected by PP concentration where the reduced disappearance in birds fed 0.34% PP diets could be the combined outcome of higher dietary PP and less available PP from rice bran (Leske and Coon, 1999; Ravindran et al., 2000).

Based on previous in vitro and in vivo work (Persson et al., 1998; Angel et al., Tamin and Angel, 2003; Tamim et al., 2004; Kim et al., 2013), reduction in IP6 and P disappearance in the distal ileum would be expected when Ca concentration increased above a phytate saturation point. Even though there was no interaction between Ca and PP, the detrimental impact of Ca was more apparent when diets containing high PP were fed, especially in the presence of phytase. For example, with increased diet Ca, disappearance of IP6 and P was reduced by only 1.2 (90.75% vs. 89.58%) and 4.7 (76.73% vs. 72%) percentage points in birds fed 0.23% PP diet with 1,000 FTU phytase/kg, respectively, whereas the reduction increased to 13.1 (86.99% vs. 73.91%) and 11.5 (70.32% vs. 58.9%) percentage points, respectively, when diet PP concentration increased to 0.34%. It is likely that in birds fed low PP diets, there was more complete removal of IPs by phytase in the gastric area of the GIT due to the lower substrate concentration and possibly source (with and without rice bran). As it is known that the binding capacity of IPs is reduced significantly once the first P was removed from IP6 (Persson et al., 1998; Yu et al., 2012), high IP6 degradation in low PP diets left very little amounts of undigested IP6 to precipitate with Ca in the lower GIT and thus a much lesser degree of reduction in IP6 or P disappearance due to increased Ca, as compared to those fed high PP diets.

Unlike in the gastric area of the GIT, where there was minimum of IP5 accumulation, a much higher increase in IP5 concentration was seen in the distal ileum when phytase-containing diets were fed to birds. The pattern of lower IP esters varied depending on phytase, Ca, and PP, but significant increases in IP3 and 4 were observed due to phytase inclusion. However, in another trial, IP4 was found to be most influenced by 500 or 1,000 FTU *Buttiauxella* phytase/kg inclusion, whereas there were no differences in ileal IP3 concentration between phytase and non-phytase treatments (Bello et al., 2016). In addition, no differences were found in ileal IP4 concentration between birds fed phytase (*A.niger* or *E. coli* phytase at 500 FTU/kg) and non-phytase diets (Zeller et al., 2015). One of the causes for the discrepancies seen in the production of IPs in the GIT may be due to differences in experimental approach (Li et al., 2014), demonstrating that broiler chickens upregulated their capacity to digest P when they were exposed to P-deficient diets for greater than 2 days. In order to avoid physiological adaptation to P deficiency, which can potentially bias phytase efficacy trials, the current trial was conducted for only 48 h, similar to the approaches taken by Tamim et al. (2004) and Li et al. (2014), which may explain the sensitivity of IPs to dietary factors.

Even though Ca adversely affects P digestion in the distal ileum, it did not affect phytase efficacy, defined as the increase in digestible P, suggesting that the majority of IP degradation happens in the upper GIT segments, where the impact of IP6 and Ca chelations is less negative to phytase efficacy (Tamim et al., 2004). Interestingly, reduced phytase efficacy was seen as a result of higher dietary PP. On average, net increase in digestible P was 17% lower in birds fed a higher PP diet. However, as discussed, this reduction could come from concentration per se and/or lower PP availability in rice bran. Across all Ca and PP, the net increase in digestible P for 500 and 1,000 FTU phytase/kg was 0.101 and 0.156%, respectively.

## CONCLUSION

It is well demonstrated that microbial phytase can effectively improve the utilization of phytate P. Results from the current study demonstrated that phytase addition significantly improved IP6 degradation in all the segments of the GIT examined in this trial. Even though negative impacts of Ca and PP were seen on ileal IP6 and P disappearance, efficacy of *Buttiauxella* phytase was primarily affected by PP concentration and possibly source. It is evident that *this* phytase can effectively degrade IP6 to lower esters, such as IP3 and IP4, but the profile of IP esters is dependent on Ca, PP, and phytase.

## Footnotes

1 Particle size and distribution for diets and limestone were determined by ASAE method S319.3, 1997.

2 Particle size distribution of low PP basal: >2.360 mm, 1.50%; 2.000-2.360 mm, 3.45%; 1.700-2.000 mm, 5.65%; 1.400-1.700 mm, 8.85%;1.180-1.400 mm; 8.65%; 1.000–1.180 mm, 9.65%; 0.850-1.000 mm, 9.65%; 0.710-0.850 mm, 7.40%; 0.500-0.710 mm, 17.25%; 0.300-0.500 mm, 14.20%; 0.250-0.300 mm, 0.65%; 0.075-0.250 mm, 6.50%; <0.075 mm, 6.60%.

3 Particle size distribution of high PP basal: >2.360 mm, 1.75%; 2.000-2.360 mm, 3.60%; 1.700-2.000 mm, 6.10%; 1.400-1.700 mm, 9.35%;1.180-1.400 mm; 9.10%; 1.000–1.180 mm, 9.70%; 0.850-1.000 mm, 10.00%; 0.710-0.850 mm, 8.75%; 0.500-0.710 mm, 17.40%; 0.300-0.500 mm, 13.10%; 0.250-0.300 mm, 0.55%; 0.075-0.250 mm, 4.75%; <0.075 mm, 5.85%.

4 Particle size distribution of limestone used in the basal and Trt diets: >1.180 mm, 0.07%; 1.000–1.180 mm, 0.71%; 0.850-1.000 mm, 5.36%; 0.710-0.850 mm, 9.74%; 0.600-0.710 mm, 10.07%; 0.500-0.600 mm, 15.96%; 0.425-0.500 mm, 10.84%; 0.355-0.425 mm, 13.10%; 0.300-0.355 mm, 9.36%; 0.250-0.300 mm, 7.88%; 0.150-0.250 mm, 10.20%; 0.075-0.150 mm, 4.99%; <0.075 mm, 1.72%.

5 Particle size distribution of monocalcium used in the basal and Trt diets: >1.180 mm, 0.82%; 1.000–1.180 mm, 8.64%; 0.850-1.000 mm, 34.83%; 0.710-0.850 mm, 24.04%; 0.600-0.710 mm, 17.22%; 0.500-0.600 mm, 9.13%; 0.300-0.500 mm, 3.81%; <0.300 mm, 1.51%.
